# Emergent Constraints on Regional Cloud Feedbacks

**DOI:** 10.1029/2021GL092934

**Published:** 2021-05-20

**Authors:** Nicholas J. Lutsko, Max Popp, Robert H. Nazarian, Anna Lea Albright

**Affiliations:** ^1^ Scripps Institution of Oceanography University of California at San Diego La Jolla CA USA; ^2^ Laboratoire de Météorologique Dynamique Ecole Normale Supérieure, Ecole Polytechnique Sorbonne Université Paris France; ^3^ Institute of Meteorology and Climatology University of Natural Resources and Life Sciences Vienna AT; ^4^ Department of Physics Fairfield University Fairfield CT USA

**Keywords:** Climate sensitivity, cloud feedbacks, emergent constraint, tropical clouds

## Abstract

Low‐cloud based emergent constraints have the potential to substantially reduce uncertainty in Earth’s equilibrium climate sensitivity, but recent work has shown that previously developed constraints fail in the latest generation of climate models, suggesting that new approaches are needed. Here, we investigate the potential for emergent constraints to reduce uncertainty in regional cloud feedbacks, rather than the global‐mean cloud feedback. Strong relationships are found between the monthly and interannual variability of tropical clouds, and the tropical net cloud feedback. These relationships are combined with observations to substantially narrow the uncertainty in the tropical cloud feedback and demonstrate that the tropical cloud feedback is likely >0Wm^−2^K^−1^. Promising relationships are also found in the 90°–60°S and 30°–60°N regions, though these relationships are not robust across model generations and we have not identified the associated physical mechanisms.

## Key Points

- Global low‐cloud‐based emergent constraints on equilibrium climate sensitivity fail in CMIP6
- Strong relationships are found between unforced cloud variability and long‐term cloud feedbacks in several regions
- Regional emergent constraints suggest the tropical cloud feedback is likely positive

## 1 Introduction

Emergent constraints are a promising tool for constraining uncertainty in Earth’s response to increased CO_2_ concentrations. The power of emergent constraints lies in relating observable variables with some aspect of the climate system’s forced response to substantially narrow the uncertainty in the projected climate response. The canonical example of an emergent constraint was proposed by Hall and Qu (2006), who demonstrated a strong correlation across climate models between the amplitude of the seasonal cycle in Northern Hemisphere snow cover and the reduction in Northern Hemisphere snow cover per degree of local warming. This strong correlation has proven to be robust across multiple climate model generations and, when combined with observations of the amplitude of Northern Hemisphere snow cover’s seasonal cycle, has allowed tight constraints to be placed on the sensitivity of Northern Hemisphere snow cover to warming (Qu & Hall, 2014; Thackeray et al., 2018).

A number of emergent constraints have been proposed for narrowing uncertainty in Earth’s equilibrium climate sensitivity (ECS), which can be broadly grouped into three categories: (1) Constraints based on historical warming rates (e.g., Flynn & Mauritsen, 2020; Jiménez‐de‐la Cuesta & Mauritsen, 2019; Nijsse et al., 2020), (2) constraints based on historical temperature variability (e.g., Cox et al., 2018; Nijsse et al., 2019), and (3) process‐based constraints, often using the variability of subtropical low clouds (e.g., Brient & Schneider, 2016; Brient et al., 2016; Lutsko & Takahashi, 2018; Qu et al., 2014; Sherwood et al., 2014; Siler et al., 2018). We focus here on the third type of emergent constraint. Several cloud‐based emergent constraints on ECS developed using CMIP5 data proposed that constraining specific cloud processes could substantially reduce uncertainty in ECS; however, when these constraints are re‐calculated using CMIP6 data the correlations between the metrics of cloud variability and models’ ECS are much lower (Schlund et al., 2020). The discrepancy in the performance of cloud‐based emergent constraints between CMIP5 and CMIP6 calls their utility into question, and suggests that temperature‐based constraints may be more fruitful approaches for constraining Earth’s ECS. Some cloud‐based emergent constraints even perform poorly when applied to CMIP5 models not included in the original analysis (Caldwell et al., 2018).

One potential explanation for why cloud‐based emergent constraints perform poorly in CMIP6 is that multiple factors are responsible for the spread in ECS across CMIP6 models. Zelinka et al. (2020) have shown that the high climate sensitivities of many CMIP6 models can be attributed in part to extratropical cloud feedbacks, including a less negative cloud feedback over the Southern Ocean, though tropical clouds still play a role. By contrast, subtropical low clouds are the main source of intermodel spread in climate feedbacks across the CMIP5 models (e.g., Andrews et al., 2012; Caldwell et al., 2016; Sherwood et al., 2014; Vial et al., 2013). If multiple cloud‐types and regions are responsible for the spread in CMIP6 models’ cloud feedback, then a single metric will struggle to constrain the global‐mean cloud feedback, and hence will struggle to constrain ECS.

These issues suggest that emergent constraints based on cloud variability cannot be used to narrow the spread of ECS among CMIP6 models, but emergent constraints on cloudiness may still be of use in more limited, local contexts. For example, an emergent constraint based on subtropical low cloud variability could be used to constrain the subtropical low cloud feedback, even if it could not be used to constrain the global‐mean cloud feedback. Similarly, new emergent constraints could be developed for the cloud feedback over the Southern Ocean. With this motivation, we propose here a new set of emergent constraints on regional cloud feedbacks. To develop these constraints, we have used the same metrics of cloud variability in each region: The regression of deseasonalized monthly surface temperature onto deseasonalized monthly cloud radiative effect (CRE, *α*_*m*_), and the regression of annual‐mean surface temperature onto annual‐mean CRE (*α*_*a*_). Using the same metrics allows us to simplify the interpretation and methodology, as new metrics do not have to be developed from scratch for each region. Instead, we can standardize the procedure for calculating the emergent constraints and using them to update the probability density functions (PDFs) of the regional cloud feedbacks. Using two predictor variables also allows us to check for consistency, as the results of emergent constraints developed with monthly variability should be consistent with the results of emergent constraints developed with interannual variability.

Taking this approach, we have investigated the links between *α*_*m*_ and *α*_*a*_ and regional cloud feedbacks in the CMIP5 and CMIP6 models. First, we demonstrate that cloud feedbacks in multiple regions contribute to the spread in CMIP6 models’ ECS, whereas tropical clouds are the primary source of spread in CMIP5 model’s ECS (Section 3). This explains the difficulty of constraining ECS in CMIP6 models using low‐cloud based emergent constraints and motivates our regional approach. We then evaluate the relationships in each region between *α*_*m*_ and *α*_*a*_, and the long‐term regional cloud feedback (Section 4). We do this for both CMIP5 and CMIP6 models to check whether viable emergent constraints are robust to the choice of models. Finally, in Section 5 we use an information‐theoretic approach to estimate posterior PDFs of the regional cloud feedbacks in those regions where strong correlations are found between the predictor variables and the regional cloud feedbacks. The posterior PDFs account for observational constraints on the regional cloud feedbacks, and our information‐theoretic approach ensures that models that are inconsistent with observations have a small influence on the posterior PDFs. Previous emergent constraint studies have often used linear regression to calculate their posterior constraints; however, given recent concerns around the reliability of emergent constraints (e.g., Caldwell et al., 2018), we believe that having multiple, complementary approaches can build confidence in and promote adoption of the results of emergent constraints.

## 2 Data and Methods

### 2.1 Observational Data

To estimate the variability of regional cloudiness in observations we have taken 17 years of monthly TOA radiative fluxes, spanning the years 2003–2019, from the Clouds and the Earth’s Radiant Energy System‐Energy Balanced and Filled (CERES‐EBAF) data set. These are matched to surface air temperatures taken from the ERA5 data set (Copernicus Climate Change Service Climate Data Store (CDS), 2017).

### 2.2 CMIP Data

Data are taken from 21 CMIP6 models and 22 CMIP5 models, listed in the supporting information. To estimate the regional cloud feedbacks, we take 500 years of data from a pre‐industrial control simulation and 150 years of data from an abrupt4XCO2 simulation with each model. The data include monthly mean values of surface air temperature, both clear‐sky and all‐sky TOA fluxes, and vertical pressure velocities at 500 hPa (see Section 4.3). To estimate *α*_*m*_ and *α*_*a*_ we use linearly de‐trended data from a historical simulation with each model, and we repeat our analyses on three non‐overlapping 17‐year segments for each set of models (1963–1980, 1980–1997, 1997–2014 for CMIP6 and 1954–1971, 1971–1988, 1988–2005 for CMIP5), then average the results.

### 2.3 Estimating Regional Cloud Feedbacks

We have calculated long‐term cloud feedbacks in five regions: 90°S–60°S, 60°S–30°S, 30°S–30°N, 30°N–60°N and 60°N–90°N. In each region, we calculate the net cloud feedback using the Gregory method (Gregory et al., 2004). First, we linearly detrend the surface temperature and net (longwave plus shortwave) CRE fields, averaged over each region, from the preindustrial control simulations, then subtract these climatological values from the 4XCO2 data. The long‐term regional cloud feedbacks are obtained by regressing the anomalous annual‐mean surface temperature onto the anomalous annual‐mean net CRE in each region for years 1–150 of the 4XCO2 simulations.

Gregory regressions are often performed for years 20–150 of 4XCO2 simulations when estimating a model’s ECS, to account for the change in slope as the global‐mean radiative feedback evolves (Andrews et al., 2015; Armour, 2017; Geoffroy et al., 2013; Winton et al., 2010). However, there are no clear changes of slope in the regional Gregory CRE plots (Figure S1), and performing the regressions for years 1–150 gives similar values to performing the regressions for years 20–150, though the uncertainties are smaller when more data are used. This is consistent with the change in the net climate feedback being caused by the evolving pattern of the surface temperature response, rather than by changes in the local feedbacks (Andrews et al., 2015; Armour et al., 2013).

We also note that the change in regional CRE per degree of regional warming is not strictly speaking the “cloud feedback,” and does not account for cloud masking of the clear‐sky response (Soden et al., 2004). Nevertheless, for ease of presentation we will refer to it as the cloud feedback hereafter.

### 2.4 Calculating Posterior PDFs of Regional Cloud Feedbacks

The goal of the emergent constraint methodology is to update the joint multi‐model prior PDF of long‐term regional feedbacks *P*_*i*_, based on the raw model data, using observational data to obtain a posterior joint multi‐model PDF *P*_*f*_. We do this following the Brient and Schneider (2016) procedure, with one notable difference.

The Brient and Schneider (2016) procedure uses an information‐theoretic distance measure between the PDFs of the observed and model regression coefficients to assign a weight *w*_*x*_ to each model *x*, where ∑_*x*_ *w*_*x*_ = 1. “Good” models, which have similar regression coefficients to the observations, are weighted more heavily, and “bad” models, whose regression coefficients are far from the observations, are given less weight. In this way, the influence of outlier models, which can exert a strong leverage on regression slopes, is minimized. We caution, however, that even “good” models may produce a close match to observations for the wrong reasons, though we are unable to account for this possibility in our framework.

The joint multi‐model PDFs *P*_*i*_ and *P*_*f*_ are calculated using Gaussian kernel density estimates. That is, as a weighted sum of the kernel value *K*_*x*_ associated with each model:

(1) $$P\left(C\right)=\underset{x}{\sum }w_{x}K_{x}\left(C\right),$$

where *C* is the long‐term cloud feedback in a given region and

(2) $$K_{x}\left(C\right)=\frac{1}{N}\underset{z=1,N}{\sum }\frac{1}{h\sqrt{2\pi }}e^{-0.5{\left(\frac{\left(C_{x}-C_{z}\right)}{h}\right)}^{2}}.$$

*N* is the number of models, *C*_*x*_ is the regional cloud feedback for model *x*, *C*_*z*_ is the regional cloud feedback for model *z* and *h* is a bandwidth parameter, set to 0.5 in all calculations, which we found gave a good compromise between smoothing the PDFs and minimizing error. The prior PDF *P*_*i*_ is calculated by assigning each model an identical weight of $w_{x}=\frac{1}{N}$, and hence does not distinguish between good or bad models.

Calculating the posterior weights requires PDFs for *α*_*m*_ and *α*_*a*_ for each climate model and for the observational data. We assume in both models and observations that the PDFs of *α*_*m*_ and *α*_*a*_ are Gaussian, and can be characterized by their mean values and standard deviations. The mean values of *α*_*m*_ and *α*_*a*_ are given by the regression coefficients of the monthly or annual regional surface temperature onto the regional CRE. The standard deviations are estimated using the standard errors of the linear regressions, which are adjusted to account for autocorrelation in the residuals (this reduces the effective sample size of the regressions). We do this by multiplying the standard errors by ([1 + *ρ*]/[1 − *ρ*])^1/2^, where *ρ* is the autocorrelation coefficient for the residuals of the regression (see Bence, 1995). Brient and Schneider (2016) used a bootstrapping procedure to estimate the standard deviations in their metric of low cloud variability, but this approach is difficult to use here because of the small number of samples for the annual‐mean data.

Together with the mean values of the regression slopes, the standard deviations are used to generate Gaussian PDFs of *α*_*m*_ and *α*_*a*_ for each model and for the observations. The model PDFs are denoted by *M*_*m*,*x*_ and *M*_*a*,*x*_ for the monthly and annual variability, respectively, and the observational PDFs are denoted by *O*_*m*_ and *O*_*a*_. Note that we calculate three sets of model PDFs, one for each 17‐year interval.

Next, we calculate the Kullback‐Leibler divergence for each model PDF:

(3) $$\Delta_{x}=\int O\left(\alpha \right)\text{log}\left(\frac{O\left(\alpha \right)}{M_{x}\left(\alpha \right)}\right)d\alpha ,$$

where we have dropped the *m* and *a* subscripts for convenience, but note that two sets of Δ_*x*_ values are calculated for each 17‐year period. Δ_*x*_ is the relative entropy between *O* and *M*_*x*_, and measures how much information is lost if *M*_*x*_ is used to approximate *O*. Importantly, this assumes the time‐series used to estimate *M*_*x*_ is the same length as the time‐series used to estimate *O*. The likelihood of model *x* giving rise to the observed distribution *O* is the exponential *l*_*x*_ = exp(−Δ_*x*_), so that normalized weights can be calculated as $w_{x}=\frac{l_{x}}{\sum_{x}l_{x}}$. Similar to weights in Bayesian model averages, the values of *w*_*x*_ can be interpreted as the posterior probability that model *x* is the best model for the data according to the Kullback‐Leibler measure (Brient & Schneider, 2016).

## 3 Sources of Intermodel Spread in ECS

**Figure 1:**
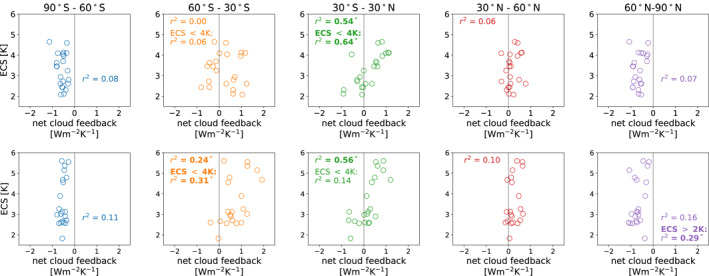
Equilibrium climate sensitivity (ECS) values of the 22 CMIP5 (top) and 21 CMIP6 (bottom) models, plotted versus the regional cloud feedbacks in the five regions. *r* ^2^ values for correlations between ECS and the regional cloud feedbacks are written in each panel, with bold values and asterisks denoting correlations with *p*‐values less than 0.05, which we take as a measure of statistical significance. The panels for 60°–30°S and 30°S–30°N also show *r* ^2^ values for correlations over models with ECS <4K, and the 60°–90°N panels show *r* ^2^ values for correlations over models with ECS >2K.

The regional cloud feedbacks, calculated as described in Section 2.3, can be used to quantify regional contributions to the intermodel spread in ECS. For example, the top row of Figure 1 demonstrates that in CMIP5 the tropical cloud feedback is highly correlated with ECS (*r* ^2^ = 0.54, all ECS values are taken from Zelinka et al., 2020), while the cloud feedbacks in all other regions are not well correlated with ECS. Hence the tropical cloud feedback is the main source of uncertainty in CMIP5 models’ ECS.

By contrast, in CMIP6 the cloud feedbacks in multiple regions are well correlated with ECS (bottom row of Figure 1; we define a correlation as statistically significant if its associated *p*‐value is less than 0.05, which corresponds to *r* ^2^ ≳ 0.2 in Figure 1). The correlation between the tropical cloud feedback and ECS again has a high *r* ^2^ value of 0.56, but the correlation between the cloud feedback in the Southern Hemisphere mid‐latitudes and ECS is also statistically significant (*r* ^2^ = 0.24). Interestingly, the Arctic cloud feedback shows a strong relationship with ECS when an outlier model (INM‐CM4‐8) which has an ECS of less than 2K, is ignored (*r* ^2^ = 0.29, note that we have included INM‐CM4‐8 in all other calculations).

To investigate these relationships further, we have divided the CMIP6 models into high sensitivity (ECS > 4K) and low sensitivity (ECS < 4K) models. Repeating the correlations, we find that the tropical cloud feedback is not well correlated with the low sensitivity models’ ECS (*r* ^2^ = 0.14, Figure 1), while the correlation with the Southern Hemisphere mid‐latitude cloud feedback is stronger for the low sensitivity models (*r* ^2^ = 0.31). The tropical and Southern Hemisphere mid‐latitude clouds feedbacks are poorly correlated among the low ECS models (not shown). Thus in CMIP6, tropical cloud feedbacks can distinguish very high climate sensitivity models from lower sensitivity models, but cannot be used to distinguish between a 2K and a 4K model. Conversely, the Southern Hemisphere mid‐latitudes can distinguish between 2K and 4K models, but are less useful for evaluating high climate sensitivities.

These results demonstrate why low‐cloud based emergent constraints perform poorly in CMIP6: A model with a large positive tropical cloud feedback likely has a high ECS, but a model with a negative tropical cloud feedback, or a tropical cloud feedback close to zero, could have an ECS of 2K or 4K. In contrast, dividing the CMIP5 models into high and low sensitivity models still gives robust relationships between tropical clouds and ECS (Figure 1).

## 4 Evaluating Regional Emergent Constraints

### 4.1 Robust Relationships

**Figure 2:**
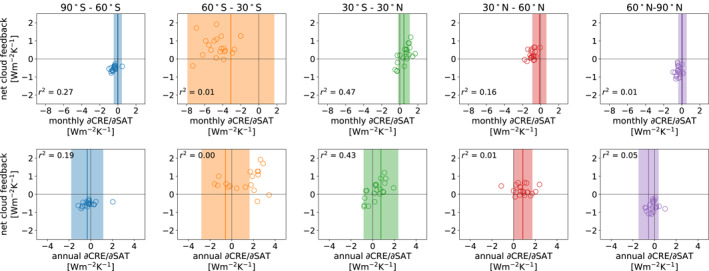
Mean values of *α*_*m*_ (top row) and *α*_*a*_ (bottom row) in the five geographic regions plotted versus the net cloud feedback in each region for 21 CMIP6 models. Only the regression coefficients calculated using the last 17 years of each historical simulation are shown. The shaded regions show 5%–95% confidence intervals for estimates of the linear regressions from Clouds and the Earth’s Radiant Energy System‐Energy Balanced and Filled data, with the solid lines showing the mean of the observational regression estimates.

We now investigate the relationships between our metrics of cloud variability and the regional cloud feedbacks. There are several robust relationships between *α*_*m*_ and *α*_*a*_ and the regional cloud feedbacks. Most notably, the regression coefficients for both monthly and interannual variability in the tropics (30°S–30°N) are highly correlated with the tropical cloud feedback in both sets of models (Table 1; Figure 2; Figure S2). Other notable relationships are seen for the 90°–60°S region in CMIP6, and the 30°–60°N region in CMIP5, though in these cases two out of the three correlations are statistically significant, while the *p*‐value for the third correlation is just over the 0.05 threshold.

**Table 1:** r^2^ Values for Correlations Across the Models Between α_m_ or α_a_ in Each Region and the Long‐Term Regional Cloud Feedbacks

Region	17‐year *α*_*m*_	17‐year *α*_*a*_	50‐year *α*_*m*_	50‐year *α*_*a*_
CMIP6				
90°S–60°S	**0.25**/0.19/**0.27**	0.12/0.10/0.19	**0.23**	**0.19**
60°S–30°S	0.08/0.08/0.01	0.08/0.08/0.00	**0.31**	**0.34**
30°S–30°N	**0.37**/**0.60**/**0.47**	**0.28**/**0.50**/**0.43**	**0.44**	**0.59**
30°N–60°N	0.11/0.11/0.16	0.04/**0.21**/0.01	**0.20**	0.08
60°N–90°N	0.05/0.07/0.01	0.03/0.10/0.05	0.0	0.02
CMIP5				
90°S–60°S	0.0/0.0/0.0	0.18/0.02/0.07	0.14	**0.32**
60°S–30°S	0.0/0.0/0.01	0.03/0.18/**0.29**	0.10	**0.34**
30°S–30°N	**0.47**/**0.35**/**0.51**	**0.59**/**0.42**/**0.36**	**0.64**	**0.67**
30°N–60°N	0.15/**0.27**/**0.26**	0.03/**0.28**/0.17	**0.35**	**0.30**
60°N–90°N	0.02/0.0/0.0	0.04/0.08/0.0	0.00	0.00
Joint				
90°S–60°S	0.35/0.02/0.04	**0.14**/0.05/**0.16**	**0.16**	**0.16**
60°S‐30°S	0.01/0.00/0.00	**0.14**/0.01/**0.16**	**0.14**	**0.38**
30°S–30°N	**0.48**/**0.40**/**0.43**	**0.50**/**0.42**/**0.41**	**0.52**	**0.64**
30°N–60°N	**0.15**/**0.22**/**0.22**	0.00/**0.21**/0.06	**0.28**	**0.21**
60°N–90°N	0.00/0.00/0.00	0.00/0.06/0.03	0.00	0.00

Columns 2 and 3 show three sets of values, one for each 17‐year period of the historical simulations. Columns 4 and 5 show correlations when *α*_*m*_ and *α*_*a*_ are estimated using the last 50 years of each simulation. Correlations with a *p*‐value less than 0.05, which we use as a measure of statistical significance, are in bold.

The observed *α*_*m*_ values for the 30°–60°N region are outside the intermodel spread in CMIP5 (Figure S2), implying that all models struggle to simulate cloud variability in this region and that we should be cautious about using this relationship to update the regional cloud feedback. Nevertheless, the observations and implied relationship do suggest that the regional cloud feedback in the 30°–60°N region is more positive than is simulated by the models. For the 90°–60°S region, there is one outlier CMIP6 model (CNRM‐CM6‐1) which is far from the observations and from the other models. Disregarding this model has a small effect on correlation between *α*_*m*_ and the regional cloud feedback (not shown), but our methodology will anyways assign a small weight to this model when calculating the posterior PDF.

As another test of the robustness of these relationships, we have taken correlations across the joint ensemble of CMIP5 and CMIP6 data. The *r* ^2^ values of these correlations are consistent with the findings from the individual ensembles (third set of rows in Table 1), with the exception of the 90°–60°S region, for which the high correlations found in CMIP6 disappear in the joint ensemble. This is not surprising, since the correlations in this region are very low in CMIP5, but suggests further caution.

### 4.2 Using Longer Time‐Series

17 years of observational data is a short record with which to establish robust correlations, but the methodology used to calculate the posterior PDFs requires that the model and observational time‐series have the same lengths. To investigate whether stronger relationships emerge with longer datasets, we have also calculated the variability coefficients *α*_*m*_ and *α*_*a*_ using the last 50 years of the historical simulations (1964–2014 in CMIP6 and 1955–2005 in CMIP5). Correlating these new coefficients with the regional cloud feedbacks gives stronger relationships than the 17 years coefficients (Table 1; Figures S3 and S4), with statistically significant relationships between *α*_*m*_ and/or *α*_*a*_ and the cloud feedbacks in all regions except for the high northern latitudes (60°–90°N).

The strong correlations for the 60°S–30°S region are of particular interest, as the Southern Hemisphere mid‐latitudes have been identified as one of the causes of the high climate sensitivities in certain CMIP6 models (Zelinka et al., 2020). The low correlation for the *α*_*m*_ in CMIP5 is due to an outlier model (see Figure S4). The calculations in Section 3 further demonstrate the importance of this region for the spread in ECS among CMIP6 models. However, the results of Section 4.1 demonstrated that the relationships between monthly/interannual variability of surface temperature and CRE in the Southern Hemisphere mid‐latitudes cannot be robustly identified from 17 years of observational data, so we cannot use observations and the methodology described in Section 2.4 to constrain the cloud feedback in this region. Moreover, the large observational uncertainty in this region suggests that emergent relationships are unlikely to be of practical use for constraining the 60°S–30°S cloud feedback in the near future, even with other methodologies.

### 4.3 Explaining the High Correlations in the Tropics

Emergent constraints are sometimes criticized as being the result of data mining (Caldwell et al., 2014, 2018; Hall et al., 2019), with no physical basis for the proposed relationships. Here, our starting assumption is that the intermodel spread in cloud physics is time‐scale invariant (note that we are not assuming the cloud physics itself is invariant, but that the causes of intermodel spread are invariant). This assumption is reasonable in the tropics, where previous emergent constraints have linked the variability of specific tropical and subtropical clouds to the net cloud feedback (e.g., Brient & Schneider, 2016; Lutsko, 2018; Zhai et al., 2015). Moreover, our results demonstrate that the unforced variability of the tropical‐mean cloud feedback, which includes contributions from all tropical cloud‐types, is related to the forced tropical‐mean cloud feedback. This suggests that the same clouds are responsible for intermodel spread in the variability and in the cloud feedback.

**Figure 3:**
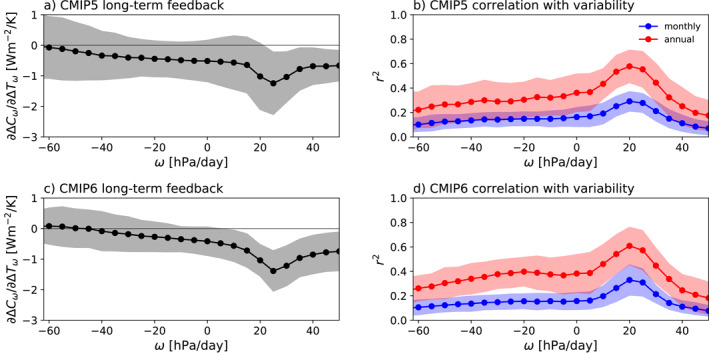
(a) Long‐term CMIP5 tropical cloud feedback in *ω*_500_ bins, calculated following Bony and Dufresne (2005) by dividing the long‐term tropical net cloud radiative effect (CRE) trend in each 5 hPa bin over years 1–150 of abrupt4XCO2 simulations by the long‐term surface temperature trend in each bin. The black markers show the multi‐model mean values and the gray shading shows ±1 standard deviation. (b) *r* ^2^ values for correlations in the CMIP5 models between the monthly (blue) and annual‐mean (red) CRE in each 5 hPa bin and the tropical‐mean CRE over the final 50 years of the historical simulations. The markers show the multi‐model mean values and the shadings show ±1 standard deviation. (c) Same as panel a but for CMIP6 models. (d) Same as panel b but for CMIP6 models.

To confirm that the same clouds drive intermodel spread in tropical CRE variability and in the tropical cloud feedback, we have binned the net CRE and surface temperature values based on the corresponding pressure velocities at 500 hPa (*ω*_500_), which is an effective method for separating out different cloud regimes in the tropics. Deep clouds and their anvils tend to dominate the CRE in regions of large‐scale ascent and low clouds tend to dominate the CRE in regions of large‐scale descent (Bony & Dufresne, 2005; Bony et al., 2004). The left panels of Figure 3 show the tropical cloud feedback in each *ω*_500_ bin, and the right panels show correlations between the monthly/annual variability of tropical net CRE in each *ω*_500_ bin and the monthly/annual variability of tropical‐mean net CRE over the historical simulations. Clouds in regimes of weak‐to‐moderate descent clearly make the largest contributions to the tropical cloud feedback (left panels) and also have the highest correlations with the tropical‐mean CRE (right panels), consistent with the large statistical weight of these subtropical low clouds (Bony & Dufresne, 2005). Hence in both sets of models, our simple metrics of tropical cloud variability mostly reflect the contributions of low clouds to monthly and interannual cloud variability, and these clouds are also the main source of uncertainty in the long‐term tropical cloud feedback.

These results are consistent with Lutsko (2018), who showed that (in models) the variations in tropical CRE during the ENSO cycle are mostly due to low clouds, with high and mid‐level clouds making minor contributions. So, while high and mid‐level clouds may show substantial differences in spatial organization on monthly, annual and ENSO time‐scales, they make relatively small contributions to the variability of the tropical‐mean radiation budget.

The physical mechanisms linking variability in other regions and the regional cloud feedbacks are less clear, and may be more difficult to identify, given the larger seasonal cycles at higher latitudes. We leave it to future work to identify the mechanisms, but note again that the results for 90°–60°S and 30°–60°N should be taken with caution until physical mechanisms can be identified.

## 5 Constraining Regional Cloud Feedbacks

**Figure 4:**
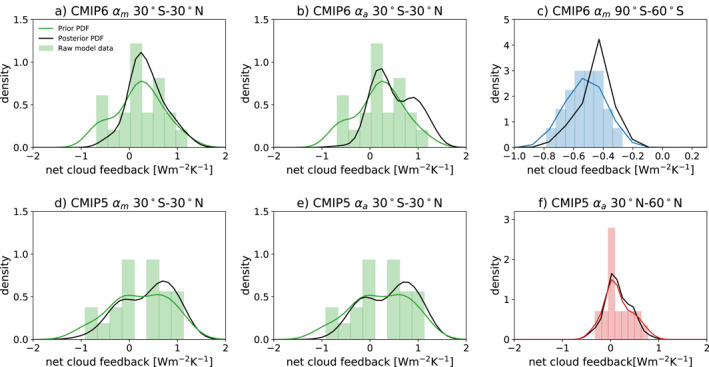
(a) Prior and posterior probability density functions (PDFs) of the tropical cloud feedback in CMIP6. The green bars show the raw model distribution of tropical cloud feedbacks and the green curves show the prior PDFs estimated using Gaussian kernel estimates. The black curves show the posterior PDFs obtained using monthly variability, following the procedure described in Section 2.4. (b) Same as panel a but the posterior PDF is obtained using interannual variability. (c) Prior and posterior PDFs of the cloud feedback in the 90°–60°S region in CMIP6. The blue bars show the raw model distribution of regional cloud feedbacks and the blue curves show the prior PDFs estimated using Gaussian kernel estimates. The black curves show the posterior PDFs obtained using monthly variability, following the procedure described in Section 2.4. (d) Same as panel a but for the CMIP5 models. (e) Same as panel b but for CMIP5 data. (f) Prior and posterior PDFs of the cloud feedback in the 30°–60°N region in CMIP5. The red bars show the raw model distribution of regional cloud feedbacks and the red curves show the prior PDFs estimated using Gaussian kernel estimates. The black curves show the posterior PDFs obtained using monthly variability, following the procedure described in Section 2.4.

Section 4 established the existence of robust relationships between the variability of tropical cloudiness on monthly and interannual time‐scales, and the long‐term tropical cloud feedback. Statistically significant relationships were also found in the CMIP6 models between the monthly variability of cloudiness and the regional cloud feedback at 90°–60°S and in CMIP5 between the monthly variability of cloudiness and the regional cloud feedback at 30°–60°N, though these relationships are less robust, particularly since they are only found in one generation of models. Using the procedure described in Section 2.4, we have estimated posterior PDFs for the cloud feedbacks in the three regions, with the results shown in Figure 4 (the posterior weights are listed in Tables S1 and S2).

In both sets of models, the monthly and interannual results for the tropics are very similar, and the posterior PDFs are consistently weighted more heavily toward positive values than the prior PDFs. This is particularly true in the CMIP6 models, where the posterior PDFs are considerably narrower: In CMIP6 the 5%–95% confidence intervals go from −0.65 to 1.26 Wm^−2^/K in the prior PDF to −0.02–1.48 Wm^−2^/K in the posterior PDF obtained using annual data or −0.09–1.29 Wm^−2^/K in the posterior PDF obtained using monthly data. In CMIP5 the 5%–95% confidence intervals narrow from −0.77 to 1.38 Wm^−2^/K in the prior PDF to −0.35–1.40 Wm^−2^/K in the posterior PDF obtained using annual data or −0.42–1.37 Wm^−2^/K in the posterior PDF obtained using monthly data. The shifts of the posterior PDFs toward more positive values are consistent with other lines of evidence pointing to a positive tropical cloud feedback (Klein et al., 2018; Myers & Norris, 2016; Scott et al., 2020; Sherwood et al., 2020). We have not investigated why the posterior PDFs are narrower when using the CMIP6 data than when using the CMIP5 data, but note that the distribution of tropical cloud feedbacks in CMIP5 is more bimodal than in CMIP6, with maxima close to 0 Wm^−2^/K and near 0.8 Wm^−2^/K. The posterior PDFs retain this bimodality, but with more weight on the maximum at 0.8 Wm^−2^/K.

For the other two regions, the posterior PDF for 90°S–60°S has a peak at around −0.4 Wm^−2^/K and is substantially narrower than the prior; while the posterior PDF for 30°–60°N is only slightly narrower than the prior. Thus an emergent constraint based on the monthly variability at 90°S–60°S has the potential to strongly constrain the cloud feedback in this region, though more work is needed to confirm this result. It will be difficult to use emergent constraints for the feedback at 30°–60°N since the models do a poor job at reproducing the variability in this region (see Figure S2).

## 6 Conclusion

The results presented here demonstrate that both the monthly and the interannual variability of cloudiness in the tropics can be used to constrain the tropical cloud feedback, with CMIP5 and CMIP6 results suggesting that the tropical cloud feedback is on the higher end of the intermodel range, and likely greater than zero. This is consistent with recent work using cloud‐controlling factors to constrain the tropical cloud feedback (Klein et al., 2018; Myers & Norris, 2016; Scott et al., 2020). At higher latitudes, we have tentatively shown that emergent constraints can be applied to the regional cloud feedbacks at 90°–60°S and 30°–60°N; with the variability in the 90°–60°S region showing particular promise as the basis for an emergent constraint. However, the high correlations between the cloud variability and cloud feedbacks in these regions are not robust across both generations of models, and we have not identified the physical mechanisms responsible for the relationships.

Another factor which limits the effectiveness of cloud‐based emergent constraints is the relatively short length of the satellite record (∼17 years). Using 50 years of model data, we have found statistically significant relationships between cloud variability and regional cloud feedbacks in all regions except 60°–90°N. These stronger correlations hint that the cloud feedback in the Southern Hemisphere mid‐latitudes (60°–30°S), a key region for the high climate sensitivities of CMIP6 models, could be constrained using the local unforced variability. Unfortunately, our metrics of variability have the highest observational uncertainty in this region, and more data will be needed before emergent constraints can be used to constrain the cloud feedback in the Southern Hemisphere mid‐latitudes. Other approaches, for example which focus on the simulation of specific cloud properties (e.g., Ceppi et al., 2016), may be more successful moving forward.

Cloud‐based emergent constraints developed in CMIP5 consistently indicated ECS is on the higher end of the intermodel range (3°–4°C, see Bretherton and Caldwell (2020)), in contrast to recent temperature‐based emergent constraints which generally suggest lower ECS values (2°–3°C; e.g., Cox et al., 2018; Nijsse et al., 2020). Reconciling these two opposing lines of evidence is of crucial importance for improving our confidence in ECS estimates. While the failure of cloud‐based emergent constraints in CMIP6 does not rule out the possibility of high ECS values, it does suggest that a more nuanced approach, moving cloud‐type by cloud‐type and region‐by‐region, will be required to reduce uncertainty in Earth’s cloud feedback.

## Supporting information

Supporting Information S1

## Data Availability

The CMIP5 and CMIP6 data are publicly available at https://esgf-node.llnl.gov/projects/esgf-llnl/, the ERA5 data are publicly available at https://www.ecmwf.int/en/forecasts/datasets/reanalysis-datasets/era5 and the CERES‐EBAF data are publicly available from https://ceres.larc.nasa.gov/data/. Jupyter notebooks with the analysis and processing scripts are available at Lutsko (2021).
