# Supplementary material for: Emergent Constraints on Regional Cloud Feedbacks
Source: Geophys Res Lett. 2021 May 20;48(10):e2021GL092934. doi: 10.1029/2021GL092934 (PMC8243946; doi:10.1029/2021GL092934)
Supplement: Supplementary file 1 — Supporting Information S1 [file GRL-48-e2021GL092934-s001.pdf]

Supporting Information for "Emergent Constraints on Regional Cloud Feedbacks"

N. J. Lutsko,[1]

Max Popp,[2]

Robert H. Nazarian,[3]

Anna Lea Albright,[2]

Corresponding author: N. J. Lutsko, Scripps Institution of Oceanography, University of California at San Diego, La Jolla, California (nlutsko@ucsd.edu)

[1]Scripps Institution of Oceanography, University of California at San Diego, La Jolla, California.

[2]Laboratoire de Météorologique Dynamique Dynamique, Sorbonne Université, Ecole Normale Supérieure, Ecole Polytechnique, Paris, France.

[3]Department of Physics, Fairfield University, Fairfield, Connecticut.

Contents of this file

1. Figures S1 to S4
2. Tables S1 and S2

Introduction The supplementary material contains four figures and 2 tables. The first figure shows an example of the the regional "Gregory" plots which are used to estimate the regional cloud feedbacks. The second figure repeats Figure 2 of the main text, but shows the results for the CMIP5 models. Supplemental Figures S3 and S4 repeat Figure 2 of the main text, but show the results when α_m and α_a are calculated using the last 50 years of each historical simulation, rather than the last 17 years. Supplemental Figure S3 shows the results for the CMIP6 models and Supplemental Figure S4 shows the results for the CMIP5 models. Table S1 shows the weights used to generate the posterior PDFs for the CMIP5 data and Table S2 shows the weights used to generate the posterior PDFs for the CMIP6 data.

The CMIP5 models used in the analysis are: BNU-ESM, CanESM2, CNRM-CM5, CSIRO-Mk3-6-0, GFDL-CM3, GFDL-ESM2G, GFDL-ESM2M, GISS-E2-H, GISS-E2-R, HadGEM2-ES, INM-CM4, IPSL-CM5A-LR, IPSL-CM5A-MR, IPSL-CM5B-LR, MIROC5, MIROC-ESM, MPI-ESM-LR, MPI-ESM-MR, MPI-ESM-P, MRI-CGCM3, NCAR-CCSM4, NorESM1-M.

The CMIP6 models used in the analysis are: BCC-CSM2-MR, BCC-ESM1, CanESM5, CNRM-CM6-1, CNRM-ESM2-1, FGOALS-f3-L, GFDL-CM4, GFDL-ESM4, GISS-E2-1-G, GISS-E2-1-H,HadGEM3-GC31-LL,INM-CM4-8, IPSL-CM6A-LR, MIROC6, MIROC-ES2L, MPI-ESM1.2-HR,MRI-ESM2-0, NCAR-CESM2, NCAR-CESM2-WACCM, NorESM2-LM, UKESM1-0-102LL. Note that the required data for

the ω_{500} binning in Figure 3 of the main text were not available for the following models at the time of the analysis (January 2021): UKESM1-0-LL, MIROC-ES2L, INM-CM4-8, NorESM2-LM, MPI-ESM1-2-HR, MIROC6 and FGOALS-f3-L.

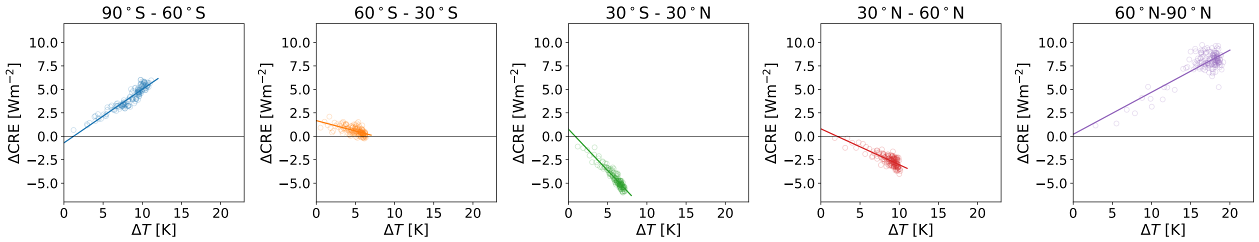

Figure S1. Example of regional "Gregory" CRE plots for the CanESM5 model. The solid lines show linear least-squares regressions to the annual-mean data shown by the open circles.

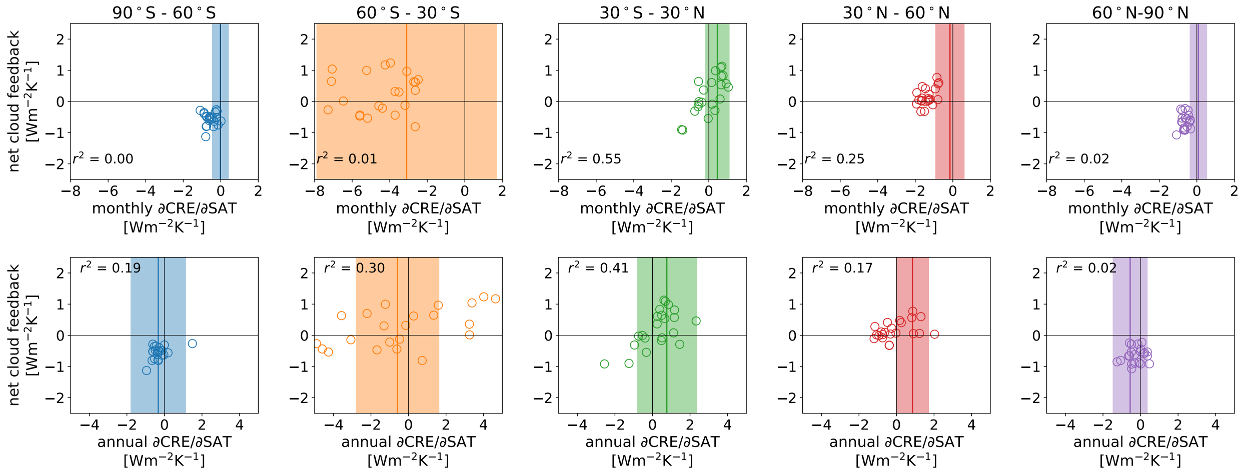

Figure S2. Values of monthly and annual linear regressions of CRE on surface temperatures in the five geographic regions plotted versus the net cloud feedback in each region for 23 CMIP5 models. The regression coefficients are calculated using the last 17 years of the historical simulations. The shaded regions show 5-95% confidence intervals for estimates of the linear regressions from CERES-EBAF data, with the solid lines showing the mean of the observational regression estimates.

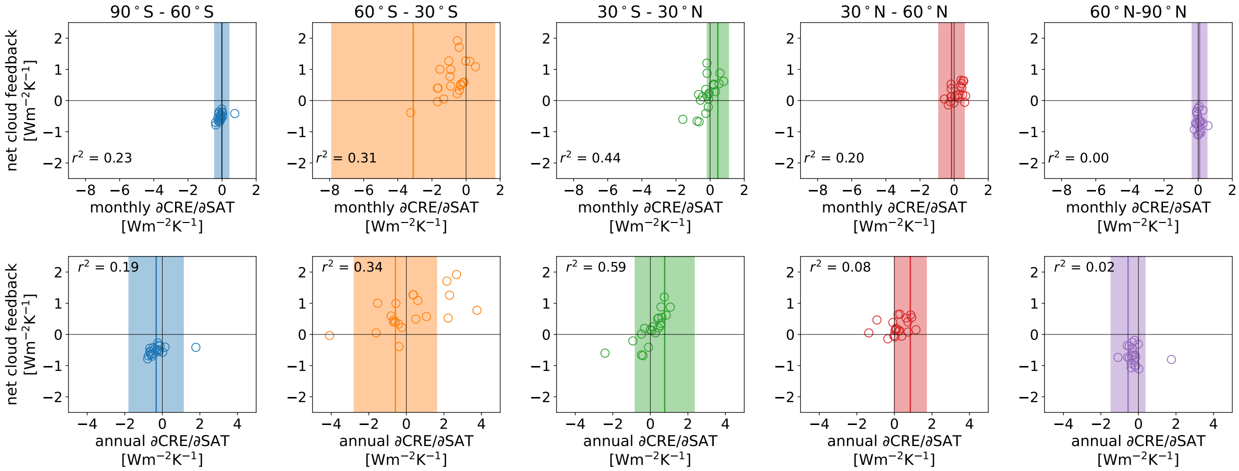

Figure S3. Same as Figure 2 in the main text, but the regression coefficients are calculated using the last 50 years of data in the historical simulations.

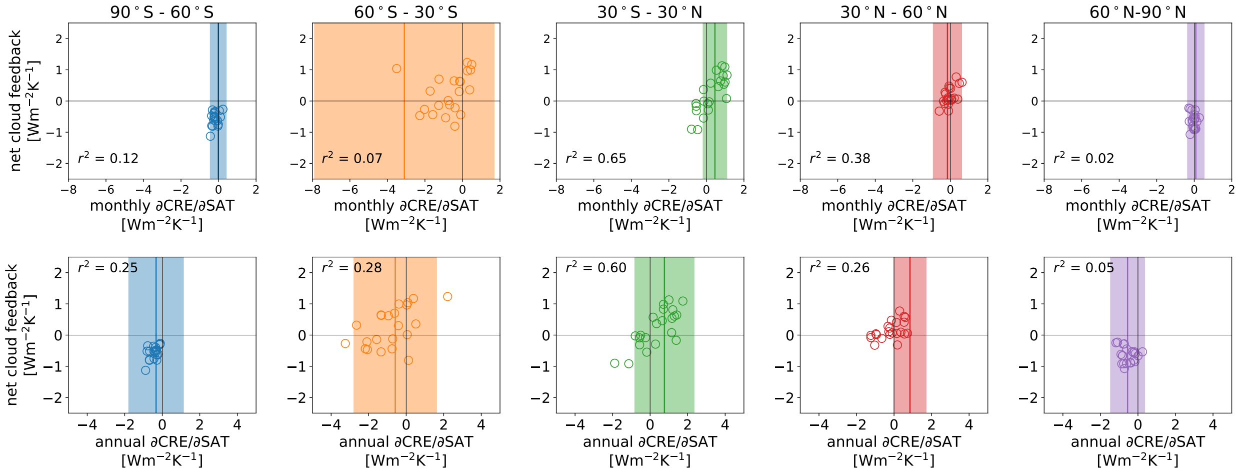

Figure S4. Same as Supplementary Figure S2, but the regression coefficients are calculated using the last 50 years of data in the historical simulations.

Table S1. Weights (in %) used to generate the posterior PDFs for the CMIP5 models. Weights are given for each 17-year segment, and the final posterior PDFs are obtained by averaging the three posterior PDFs obtained from each 17-year segment.

Model	α_m 30°S-30°N	α_a 30°S-30°N	α_m 30°-60°N
BNU-ESM	1.0/8.4/3.8	2.0/9.2/2.6	0.2/0.0/0.0
CanESM2	0.5/0.0/8.7	5.5/0.1/9.7	12.2/9.3/1.1
CNRM-CM5	0.1/0.0/0.0	0.1/0.2/0.5	8.1/3.8/0.0
CSIRO-Mk3-6-0	6.9/8.7/6.1	9.0/6.6/5.8	0.0/0.0/11.2
GFDL-CM3	15.9/2.8/9.6	8.4/5.6/8.7	10.9/4.0/7.9
GFDL-ESM2G	0.0/0.0/0.0	1.6/1.2/0.0	11.9/8.9/0.2
GFDL-ESM2M	0.0/11.7/0.9	0.0/8.1/0.7	8.8/0.1/14.5
GISS-ESM-H	0.0/0.0/0.0	0.0/0.0/0.0	0.6/0.0/1.2
GISS-ESM-R	0.7/4.6/0.0	0.6/0.4/0.0	10.6/0.5/0.0
HADGEM2-ES	11.5/9.2/7.5	11.9/7.8/8.8	1.5/5.5/8.6
INMCM4	8.5/2.0/8.0	8.6/5.0/8.8	11.4/14.1/12.6
IPSL-CM5A-LR	0.1/0.0/8.4	1.1/0.0/9.8	2.2/1.1/0.0
IPSL-CM5A-MR	8.2/0.0/9.0	11.4/2.1/9.6	1.5/0.2/0.5
IPSL-CM5B-LR	13.8/0.4/8.8	1.1/5.9/0.6	8.4/0.0/0.0
MIROC5	13.7/6.5/1.2	9.3/4.5/6.6	0.0/1.5/11.1
MIROC-ESM	4.7/8.0/4.0	9.6/7.5/3.7	0.0/0.2/0.1
MPI-ESM-LR	0.0/13.3/0.2	0.0/8.4/0.0	0.2/12.1/1.7
MPI-ESM-MR	0.5/5.4/8.7	0.2/7.8/2.2	10.6/3.4/0.0
MPI-ESM-P	1.3/4.9/0.1	7.8/2.7/3.7	0.0/5.8/4.0
MRI-CGCM3	12.2/2.0/4.5	9.5/5.4/8.9	0.3/0.0/1.7
NCAR-CCSM4	0.1/4.3/10.2	0.8/4.9/8.9	0.5/8.8/13.6
NorESM1-M	0.1/7.8/0.3	1.6/6.6/0.5	0.1/20.7/9.7

Table S2. Weights (in %) used to generate the posterior PDFs for the CMIP6 models. Weights are given for each 17-year segment, and the final posterior PDFs are obtained by averaging the three posterior PDFs obtained from each 17-year segment.

Model	α_m 30°S-30°N	α_a 30°S-30°N	α_m 60°-90°S
BCC-CSM2-MR	1.2/7.2/1.9	9.1/4.5/7.8	4.2/2.7/7.6
BCC-ESM1	0.0/6.2/0.5	3.1/10.5/8.6	0.6/14.7/0.8
CanESM5	10.2/9.2/3.9	12.7/12.0/10.0	1.2/4.4/6.5
CNRM-CM6-1	1.5/7.8/10.5	0.5/1.1/0.0	64.0/26.0/24.1
CNRM-ESM2-1	12.3/5.9/5.7	0.0/2.1/1.1	11.9/3.8/0.4
FGOALS-f3-L	5.6/0.0/0.4	0.0/0.0/0.0	0.0/0.0/0.0
GFDL-CM4	3.1/7.2/9.9	7.2/4.7/9.2	0.1/0.0/0.0
GFDL-ESM4	5.0/10.6/4.5	5.0/0.1/8.1	0.0/1.3/0.9
GISS-E2-1-G	0.0/0.0/0.0	0.1/0.0/0.0	0.0/0.0/3.2
GISS-E2-1-H	0.0/0.0/0.0	0.0/0.3/0.0	2.3/0.3/1.5
HadGEM3-GC31-LL	3.9/3.5/7.7	0.0/0.2/6.0	5.4/9.2/12.5
INM-CM4-8	0.0/0.0/0.3	0.0/0.1/2.7	2.0/0.1/2.6
IPSL-CM6A-LR	9.8/8.9/8.7	10.6/5.9/0.9	4.1/25.1/2.1
MIROC6	12.8/7.7/10.8	9.7/10.0/0.7	0.0/0.0/0.0
MIROC-ES2L	5.6/0.0/8.2	0.0/0.0/0.0	0.0/0.0/0.0
MPI-ESM1.2-HR	1.5/0.2/6.9	0.4/2.1/9.6	0.7/0.9/0.3
MRI-ESM2-0	8.2/4.6/3.7	4.6/2.6/0.9	0.5/3.7/4.8
NCAR-CESM2	6.7/3.4/5.8	13.1/12.2/11.6	0.6/0.5/0.8
NCAR-CESM2-WACCM	5.6/4.4/3.7	13.1/11.8/14.1	1.2/2.6/1.0
NorESM2-LM	6.1/6.7/6.9	10.3/11.5/6.9	0.4/4.0/29.6
UKESM1-0-102LL	0.8/6.3/0.0	0.5/10.6/1.9	0.7/0.7/1.2
